# Video Coding Based on Ladder Subband Recovery and ResGroup Module

**DOI:** 10.3390/e27070734

**Published:** 2025-07-08

**Authors:** Libo Wei, Aolin Zhang, Lei Liu, Jun Wang, Shuai Wang

**Affiliations:** 1 School of Mathematics and Information Science, Hebei University, Baoding 071000, China; libowei2024@163.com (L.W.); zhangaolin5743@163.com (A.Z.); 2 School of Computer Science and Technology, Huaibei Normal University, Huaibei 235000, China; wangshuai@chnu.edu.cn; 3 Huaibei Key Laboratory of Digital Multimedia Intelligent Information Processing, Huaibei 235000, China; 4 College of Electronic and Information Engineering, Hebei University, Baoding 071000, China; junwanghbu@hbu.edu.cn

**Keywords:** video coding, discrete wavelet transform (DWT), image reconstruction, attention mechanism, ResGroup

## Abstract

With the rapid development of video encoding technology in the field of computer vision, the demand for tasks such as video frame reconstruction, denoising, and super-resolution has been continuously increasing. However, traditional video encoding methods typically focus on extracting spatial or temporal domain information, often facing challenges of insufficient accuracy and information loss when reconstructing high-frequency details, edges, and textures of images. To address this issue, this paper proposes an innovative LadderConv framework, which combines discrete wavelet transform (DWT) with spatial and channel attention mechanisms. By progressively recovering wavelet subbands, it effectively enhances the video frame encoding quality. Specifically, the LadderConv framework adopts a stepwise recovery approach for wavelet subbands, first processing high-frequency detail subbands with relatively less information, then enhancing the interaction between these subbands, and ultimately synthesizing a high-quality reconstructed image through inverse wavelet transform. Moreover, the framework introduces spatial and channel attention mechanisms, which further strengthen the focus on key regions and channel features, leading to notable improvements in detail restoration and image reconstruction accuracy. To optimize the performance of the LadderConv framework, particularly in detail recovery and high-frequency information extraction tasks, this paper designs an innovative ResGroup module. By using multi-layer convolution operations along with feature map compression and recovery, the ResGroup module enhances the network’s expressive capability and effectively reduces computational complexity. The ResGroup module captures multi-level features from low level to high level and retains rich feature information through residual connections, thus improving the overall reconstruction performance of the model. In experiments, the combination of the LadderConv framework and the ResGroup module demonstrates superior performance in video frame reconstruction tasks, particularly in recovering high-frequency information, image clarity, and detail representation.

## 1. Introduction

### 1.1. Current Research Status of Video Coding

The high efficiency video coding (HEVC) standard, released in 2012, makes a significant advancement in video coding technology as a successor to H.264. Research by Sullivan et al. [1] demonstrated HEVC’s substantial improvement in compression efficiency, particularly through the introduction of fine-grained coding units (CUs), more accurate motion estimation, and complex inter-frame prediction methods, which resulted in a nearly 50% improvement in bitrate efficiency compared to H.264. This enhancement makes HEVC an ideal choice for high-resolution video encoding, such as 4K and 8K. With the increasing demand for internet video streaming, the AV1 [2] standard, as a successor to HEVC and VP9, has performed excellently. AV1 provides higher compression efficiency, improving compression efficiency by about 30% over HEVC for the same video quality. Its open-source nature also resolves the patent licensing issues associated with HEVC, earning support from many internet giants and gradually becoming the mainstream video coding format. Furthermore, the versatile video coding (VVC) [3] standard achieves significant bitrate reduction of 50% over HEVC standard, and of 75% over the advanced video coding (AVC) standard, due to several new technologies such as enhanced coding tree unit partitioning, advanced intra- and inter-prediction, multiple transform selection, enhanced loop filters, etc. However, despite its remarkable progress in compression efficiency, the increased computational complexity and decoding delay, particularly in low-latency applications, limit its use in real-time video streaming and low-latency scenarios.

With the rise of deep learning technology, the video coding field has gradually begun exploring new optimization paths. Jacob et al. [4] proposed a deep learning-based video compression method using convolutional neural networks (CNNs). This method dynamically optimizes compression strategies through feature extraction and adaptive learning, learning efficient compression features from large amounts of video data, significantly improving video compression efficiency and quality at low bitrates. The potential of deep learning in video coding has become increasingly evident, and the end-to-end video coding method proposed by Rippel et al. [5] has further driven this development. By combining deep neural networks, end-to-end coding methods can dynamically adjust encoding strategies based on video content and network conditions, avoiding excessive reliance on predefined parameters in traditional coding frameworks. This makes video coding more flexible, particularly excelling in real-time video streaming and communication scenarios.

In response to the demand for low-latency video coding, Jedari et al. [6] proposed a coding scheme optimized for motion estimation and prediction mechanisms, specifically targeting live streaming and real-time video transmission. This scheme reduces the delay in video coding while maintaining high video quality, offering new ideas for high-resolution, low-latency video streaming. Lu et al. [7] proposed an end-to-end video coding method based on deep learning, which further deepens this trend. By training a neural network directly from raw video to compressed video output, this method optimizes every step of the compression process and dynamically adjusts compression strategies based on different video content and network conditions. This innovation not only improves compression efficiency but also maintains high video quality in low-bandwidth environments. Meanwhile, deep video super-resolution and denoising technologies have also become hot research topics. Bao et al. [8] proposed a video denoising method based on CNN that can recover lost details under low resolution and high compression ratios, significantly improving video clarity and visual effects. These technologies are not only applicable to video transmission in low-bandwidth environments but also enhance the user experience in high-definition video streaming and live broadcasting.

At the same time, AV1 encoder performance optimization is also advancing. Kyslov et al. [9] proposed a method to improve parallel processing techniques and inter-frame compression algorithms, significantly enhancing AV1’s compression speed and decoding performance in real-time applications, particularly in low-latency and high-frame-rate video streaming and live broadcasting applications. As a result, AV1 has become a more practical video coding format, especially suitable for high-quality, low-latency video transmission. With ongoing technological progress, Chen et al. [10] proposed a multimodal deep learning-based video coding method. This approach integrates information from different modalities, such as visual, speech, and metadata, further improving video coding efficiency and quality. It not only dynamically adjusts compression strategies but also optimizes compression methods based on video scenes and context, thereby reducing data transmission while maintaining high visual quality. This innovation provides new ideas for the development of intelligent video coding systems, showing broad applicability in multi-scene and multimedia data stream applications. Medeiros et al. [11] proposed an adaptive video streaming compression technique based on deep learning end-to-end optimization methods, capable of real-time adjustment of compression rates and video quality according to network bandwidth and video content changes. This technique significantly reduces stuttering and quality fluctuations in video streams in unstable network environments, improving video transmission efficiency and offering broad application prospects, especially for streaming services, online education, and virtual reality.

More recently, diffusion model and representation learning based video generation and editing have attracted a lot of attention. Blattmann et al. [12] proposed a scaling latent video diffusion model for text-to-video and image-to-video generation, which comprises three main stages of text-to-image pretraining, video pretraining, and high-quality video finetuning. The stable video diffusion model achieves high-resolution and high coherence video generation on large datasets. However, for video editing, diffusion-based methods often face challenges in editing existing objects within video sequences while maintaining temporal coherence in their appearance. Chai et al. [13] proposed a StableVideo framework to achieve consistent video editing. The StableVideo introduces temporal consistency to generate consistent appearance for the edited objects. Also, a novel inter-frame propagation mechanism is proposed for diffusion-based video editing, which helps to transfer the layered appearance information across consecutive video frames. Yang et al. [14] proposed a diffusion transformer-based large-scale text-to-video generation model CogVideoX. By spatiotemporal compression architecture, expert level cross modal fusion, and progressive long video training, CogVideoX achieves high-resolution, long duration, and strong narrative text-to-video generation capability for the first time. Zhang et al. [15] proposed a training-free motion-guided video generation framework, which achieves high-quality video generation without additional training through innovative motion consistency loss and initial noise optimization, setting a new benchmark in time coherence and motion control. Moreover, Cai et al. [16] proposed multimodal contrastive learning (MMCL). Formalizing the cross-modal misalignment by selection bias and perturbation bias, the MMCL reconciles the perspectives of mitigating and leveraging misalignment. Liu et al. [17] explores whether large language models have the ability to achieve interpretable human concepts through simple data operations or by truly capturing them through generative models and theoretical analysis.

In summary, video coding technology has evolved from traditional coding standards (such as HEVC) to deep learning-driven innovative methods. With the continuous development of deep learning and adaptive technologies, future video coding technologies will continue to break through in compression efficiency, real-time performance, and adaptability to meet the growing demand for video streaming and low-latency application scenarios. As multimodal information is integrated, the flexibility and intelligence of video coding will also improve significantly, bringing more possibilities to various application scenarios.

### 1.2. Discrete Wavelet Transform and Deep Learning-Based Video Coding

In recent years, the combination of discrete wavelet transform (DWT) and deep learning has garnered widespread attention in the fields of video coding and image compression. The advantage of wavelet transform lies in its powerful capabilities in multi-scale, localization, and frequency-domain analysis, making it an ideal choice for improving image and video compression performance when combined with deep learning technologies. In particular, the fusion of DWT and deep learning has shown significant potential in maintaining high-quality reconstruction. Specifically, DWT can decompose images or videos into multiple frequency bands, providing a foundation for deep learning models to extract features and compress data across different bands, thereby achieving outstanding performance in compression efficiency and reconstruction quality.

In this context, Li et al. [18] proposed an innovative video coding method combining DWT with CNN. The method first decomposes video frames into multiple frequency bands using wavelet transform, and each band is then input into the CNN for feature extraction and compression. CNNs significantly improve compression efficiency by automatically extracting important features from video frames. Finally, after the inverse wavelet transform, the compressed video frames are reconstructed. Experimental results show that this method effectively preserves video quality in low-bandwidth environments and offers significant advantages in compression efficiency, making it especially suitable for low-bandwidth network applications.

Similarly, Mishra et al. [19] proposed an image compression and reconstruction method combining DWT with deep convolutional autoencoders (DCAEs). Unlike video coding, image compression requires handling finer spatial and frequency features. The method first decomposes the image into low-frequency and high-frequency components using wavelet transform, then compresses and encodes the low-frequency component using a deep autoencoder, and finally reconstructs the image through the decoder. By extracting multi-scale features from the image using DWT, and learning more compact representations with the deep autoencoder, this method effectively improves the compression ratio while maintaining high reconstruction quality. Compared to traditional JPEG methods, this approach shows significant improvements in image detail restoration and compression ratio.

In further exploration of image compression, Jia et al. [20] proposed a method combining DWT with deep CNNs for image compression. Similar to the previous method, this approach first decomposes the image using DWT, then processes the low-frequency component (which carries the main information) using CNNs, while the high-frequency component is optimized to preserve image details or reduce noise. CNNs not only efficiently compress redundant data but also delve into the local features of the image, further enhancing reconstruction quality. By extracting multi-scale features, this method better preserves details and structure during image reconstruction. Compared to traditional compression methods like JPEG, it achieves higher compression efficiency and visual quality.

In the application of generative adversarial networks (GANs), Fu et al. [21] proposed an image compression method combining DWT with GAN. Unlike previous methods, this approach uses the adversarial training mechanism of GAN to further enhance the preservation of image details and compression effectiveness. By performing frequency decomposition of the image using DWT, the GAN focuses on compressing the low-frequency components and effectively maintains image quality at lower bitrates. Compared to traditional compression methods, GAN-based image compression more efficiently preserves image details and produces superior visual results during image reconstruction.

Additionally, Dong et al. [22] proposed a video compression method combining DWT with deep neural networks. Unlike image compression, video coding not only needs to process spatial information but also involves compressing consecutive frames in the temporal domain. The method first uses DWT to perform multi-scale decomposition of video frames, then encodes the low-frequency component using a deep neural network, while the high-frequency component is compressed using traditional quantization and coding techniques. The low-frequency component typically contains the primary information of the video. Therefore, using deep learning to compress the low-frequency component not only enhances compression efficiency but also helps maintain high video quality during reconstruction. Experimental results show that this method outperforms traditional video coding standards such as H.264 and HEVC, especially in low-bandwidth network environments.

In summary, the combination of DWT and deep learning has made significant progress in the fields of video coding and image compression. By integrating multi-scale analysis with deep learning models, researchers have achieved notable improvements in both compression efficiency and reconstruction quality. Particularly in terms of detail preservation and low-bitrate compression, these methods far outperform traditional compression algorithms like JPEG, H.264, and HEVC. With the continued development of deep learning technologies, the fusion of DWT and deep learning will provide new directions for the advancement of image and video compression, driving these technologies toward broader real-world applications.

### 1.3. Contributions of This Study

With the rapid development of video coding technologies, improving the reconstruction quality of video frames, particularly the recovery of high-frequency details such as edges and textures, remains a significant challenge. Traditional methods, such as HEVC, often struggle with recovering fine details, particularly at low bitrates. This paper addresses this issue by introducing a novel LadderConv framework integrated with a ResGroup module, designed to enhance the recovery of high-frequency information, in response to the challenges of insufficient accuracy and information loss in recovering high-frequency detail information in traditional video coding methods. Specifically, the main contributions of this paper can be summarized as follows:

- Innovative LadderConv Framework: This paper introduces a framework based on a step-by-step recovery of wavelet subbands, which gradually recovers low-frequency and high-frequency information, especially detailed features (such as edges and textures), thus improving the coding quality of video frames. The framework first processes the high-frequency subbands with less information and then enhances the interaction between the subbands during the recovery process, ultimately generating high-quality reconstructed images through inverse wavelet transform. To further improve detail recovery and image reconstruction accuracy, the LadderConv framework incorporates spatial attention and channel attention mechanisms. The spatial attention mechanism effectively focuses on important spatial regions in the image, enhancing the prominence of key information; the channel attention mechanism applies weights to different channel features, improving the information expression capability, thus ensuring the quality and accuracy of the reconstructed image.
- Combination of Wavelet Transform and Step-by-Step Recovery: By combining step-by-step recovery of wavelet transforms, the LadderConv framework can leverage both low-frequency and high-frequency information to progressively restore the image details while ensuring overall reconstruction quality. The framework organically combines the low-frequency and high-frequency subbands, maintaining the integrity of the low-frequency information while improving the restoration of image details.
- Innovative ResGroup Module: To optimize the performance of the LadderConv framework in video coding tasks, particularly in detail recovery and high-frequency information extraction, this paper designs an innovative ResGroup module. Through multi-layer convolution operations, feature map compression and recovery, and feature fusion strategies, the ResGroup module not only enhances the network’s expressiveness but also effectively reduces computational complexity. The module can capture multi-level features from low to high levels and retains rich feature information through residual connections, further improving the model’s reconstruction performance.

## 2. Proposed Video Coding Framework

**Figure 1:**
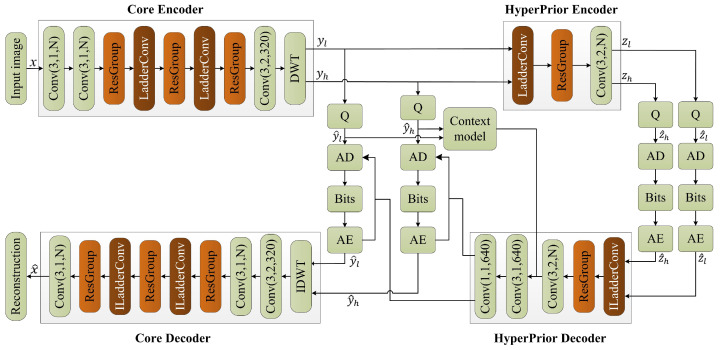
Proposed video coding framework based on stepwise recovery of wavelet subbands and residual group module.

In this section, we first introduce the video encoding framework based on the ladder subband recovery and ResGroup module, as shown in Figure 1. Then, we provide a detailed description of the LadderConv module and the ResGroup module, along with an in-depth explanation of the design of the loss function.

### 2.1. Video Coding Framework Based on DWT and ResGroup Module

#### 2.1.1. Core Encoder

The input image *x* has a size of H×W×C (height, width, and channels). It is first processed through multiple convolutional layers in the main encoder to gradually extract image features. The main encoder consists of several convolutional layers, each using a 3×1 convolution kernel, outputting *N* feature maps, aiming to capture vertical features in the image such as edges and textures.

The ResGroup module is designed to capture multi-level features through a sequence of convolutions. The input feature maps pass through two residual blocks, ResGroup1 and ResGroup2. Each block applies a series of convolutions: the first convolution 3×1 reduces the number of channels from *N* to N/2, followed by a channel compression using a 1×1 convolution. Finally, the number of channels is restored to *N* through a second 3×1 convolution. Each convolution is followed by a LeakyReLU activation, introducing non-linearity, and the output is then passed through a 1×1 convolution with a Sigmoid activation function to generate the final output feature map. After each convolution operation, a LeakyReLU activation function is applied to introduce non-linearity, and finally, a 1×1 convolutional layer and Sigmoid activation function are used to generate the output feature map $R_{1}$. The input then passes through ResGroup2, which has the same structure as ResGroup1, generating the output feature map $R_{2}$.

After processing through the two residual groups, the feature maps $R_{1}$ and $R_{2}$ are first multiplied through matrix multiplication and then element-wise added to the original input feature map $R_{in}$, generating the final output feature map $R_{out}$. This feature map merges multi-layer feature information, helping to improve the performance of the model.

The LadderConv module utilizes DWT to decompose video frames and gradually reconstruct each wavelet subband, thus improving the video frame reconstruction quality. The input video frame is first decomposed via wavelet transform to obtain low-frequency approximation coefficients and high-frequency detail coefficients. The LadderConv framework adopts a step-by-step recovery strategy, starting with the recovery of detail subbands with less information, such as diagonal, horizontal, and vertical detail coefficients, and then progressively supplementing these details to assist in the recovery of the low-frequency approximation subband. Next, the framework combines the recovered detail information with the low-frequency information and performs inverse wavelet transform to generate the reconstructed image. During this process, spatial attention and channel attention mechanisms further optimize the image reconstruction performance. The spatial attention mechanism focuses on key regions in the image, enhancing the saliency of important information, while the channel attention mechanism weights the feature responses from different channels, improving the expressiveness of the information.

In DWT, the input image is first decomposed into low-frequency approximation coefficient subband $y_{l}$ and multiple high-frequency detail coefficient subbands $y_{h}$. The low-frequency information $y_{l}$ contains the primary structure and global information of the image, representing the overall outline and smooth regions. The high-frequency information $y_{h}$ captures detail information in the image, including edges and texture features, reflecting the local variations of the image. Through this decomposition, DWT effectively separates the different frequency components of the image, providing more detailed feature information for subsequent image reconstruction and detail restoration.

#### 2.1.2. Hyper-Prior

The low-frequency information $y_{l}$ and high-frequency information $y_{h}$ obtained from the decomposition are input into the hyper-prior region for feature extraction and encoding, generating the hyper-prior information $z_{l}$ and $z_{h}$. The structure of the hyper-prior encoder consists of LadderConv, ResGroup, and a 3×2 convolution layer. The LadderConv layer performs initial feature extraction through convolution operations, enhancing the feature representation ability. The ResGroup adopts a residual block structure to further extract and fuse features, improving the model’s expressive power. A 3×3 convolution kernel with a stride of 2 is used to reduce the size of the feature maps and increase the number of channels, making it suitable for subsequent processing. First, the hyper-prior encoder receives the latent representations $y_{l}$ and $y_{h}$, and generates the hyper-prior information $z_{l}$ and $z_{h}$ through a multi-layer convolutional network:

(1) $$\left\{\begin{array}{c} z_{l}=HyperEncoder\left(y_{l}\right),\\ z_{h}=HyperEncoder\left(y_{h}\right). \end{array}\right.$$

The hyper-prior information $z_{l}$ and $z_{h}$ are then quantized to obtain the quantized hyper-prior information ${\hat{z}}_{l}$ and ${\hat{z}}_{h}$, reducing the data size and making it easier for storage and transmission. Next, the quantized feature maps are input into the arithmetic encoder (AE) for encoding.

Finally, the quantized and encoded features are decoded through the hyper-prior decoder. Specifically, the last two convolution operations are used to restore the number of channels in the feature map to 640 and output the latent representations $y_{l}$ and $y_{h}$, which represent the reconstruction results. The hyper-prior region effectively extracts and reconstructs image features, thereby enhancing the performance and quality of image processing.

#### 2.1.3. Core Decoder

The structure of the main decoder is designed to progressively restore the encoded image information to achieve high-quality image reconstruction. The decoding process first combines the low-frequency information $y_{l}$ and high-frequency information $y_{h}$ using the inverse discrete wavelet transform (IDWT), recovering the initial image features. Next, a 3×1 convolution is applied to downsample the image and extract features, further enhancing the feature representation capability.

Subsequently, the main decoder continues with a convolution layer to adjust the number of channels, restoring the feature map’s channel count to *N*, ensuring consistent feature dimensions for subsequent processing. The input feature map is then passed into a ResGroup, which further extracts and fuses feature information through multiple convolution operations and activation functions, improving the accuracy of image reconstruction.

After that, the main decoder uses the ILadderConv module, combining the strategy of inverse wavelet transform to progressively restore detail information. By integrating with the low-frequency information, it ensures that the image’s details and structure are well preserved. The output of this module is then passed through another ResGroup to further enhance the feature representation ability. In the following steps, the ILadderConv module is used again to continue refining the image information, ensuring the integrity and clarity of the details. Finally, after a convolution layer, the main decoder outputs the final reconstructed image. Through this series of processing steps, the main decoder effectively transforms the features extracted during the encoding phase into a high-quality reconstructed image, ensuring the best visual effect and recovery of details.

### 2.2. LadderConv Module

After the input video frame $V_{in}$ is decomposed using DWT, the primary information is concentrated in the approximation coefficients subband, with more information in the low-frequency part. The high-frequency part (detail information) primarily represents edge and texture features, which contain relatively less information. Specifically, the vertical detail coefficients, horizontal detail coefficients, and diagonal detail coefficients represent the high-frequency components in the vertical, horizontal, and diagonal directions, respectively, with the diagonal detail coefficients containing the least information.

**Figure 2:**
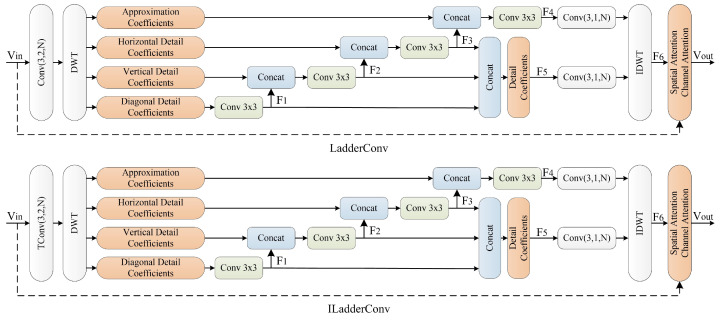
The proposed LadderConv module.

After the wavelet decomposition, each subband reflects image information from different perspectives and contains inherent relationships. Based on this, the LadderConv framework is proposed as shown in Figure 2, which adopts a stepwise order to progressively recover the wavelet subbands. The subbands with less information (diagonal detail coefficients, horizontal detail coefficients, and vertical detail coefficients) are processed first, and then the recovered subbands help restore the approximation coefficients subband with more information.

The specific process is as follows: First, the diagonal detail coefficients wavelet subband $F_{1}$ is restored, providing necessary auxiliary information for subsequent recovery. Then, the horizontal detail coefficients wavelet subband $F_{2}$ is restored. By combining with $F_{1}$, the effect of information recovery is enhanced. Finally, the vertical detail coefficients wavelet subband $F_{3}$ is restored, which, together with the previous subbands $F_{1}$ and $F_{2}$, helps recover the approximation coefficients subband $F_{4}$. Through the stepwise recovery of different subbands, the LadderConv framework effectively utilizes the information after the wavelet transform, ensuring mutual support and supplementation between the subbands.

The wavelet subbands $F_{1}$, $F_{2}$, and $F_{3}$, together, form the detail information, which is progressively restored to improve the video frame reconstruction quality and connect to form a new subband $F_{5}$. The approximation coefficients subband $F_{4}$ provides the overall low-frequency information and serves as the foundation for detail recovery. By combining the approximation coefficients subband $F_{4}$ with the detail information subband $F_{5}$ and performing an inverse wavelet transform, the reconstructed image representation $F_{6}$ is generated, ensuring high-quality image reconstruction and improving the visual effect of the video frame.

After the reconstructed image representation $F_{6}$ is obtained, the input video frame $V_{in}$ and the reconstructed image representation $F_{6}$ are further processed using spatial attention and channel attention mechanisms to enhance the effective utilization of information. The spatial attention mechanism focuses on important spatial regions of the image, improving the saliency of key information. The channel attention mechanism weights the responses of different channel features, enhancing the capability of information expression. These processing steps together generate the output video frame $V_{out}$, ensuring effective integration and enhancement of information during the reconstruction process.

Thus, the LadderConv framework progressively recovers the wavelet subbands in a stepwise manner, fully utilizing both low-frequency and high-frequency information. It restores the image from details to the overall structure, ensuring high-quality reconstruction. By organically combining detail information and approximation information, the framework enhances the restoration of image details while preserving the integrity of low-frequency information, resulting in a more refined image. Furthermore, the framework employs spatial and channel attention mechanisms, which further enhance the feature expression of key regions and channels, improving the quality and accuracy of the reconstructed image. This framework is not only applicable to static images but also to video frames, enhancing temporal detail recovery and improving edge and texture details, thereby improving the visual effect and overall quality of the video frame.

### 2.3. ResGroup Module

**Figure 3:**
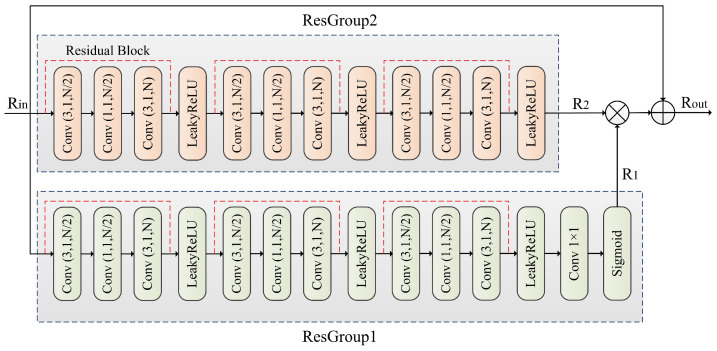
The proposed ResGroup module.

The ResGroup module enhances the capabilities of the LadderConv framework through multi-level convolution operations, feature compression and recovery, as well as feature fusion strategies. As shown in Figure 3, the ResGroup module progressively extracts and fuses low-level to high-level features, helping LadderConv more effectively recover the subbands after wavelet transformation. In particular, when handling low-frequency and high-frequency information, the ResGroup module facilitates the interaction and supplementation of features at different scales through matrix multiplication and element-wise addition fusion, thereby improving the detail recovery performance of LadderConv and enhancing the image reconstruction quality. At the same time, the ResGroup module reduces computational complexity through 1×1 convolutions and boosts the network’s expressive power through non-linear activations, further improving the overall visual effect and detail representation of the image.

The ResGroup module mainly consists of ResGroup1 and ResGroup2, matrix multiplication modules (denoted as matrix multiplication), and element-wise addition modules (denoted as element-wise addition). The ResGroup module is primarily designed to work with the LadderConv module.

ResGroup1 first uses convolution operations for feature extraction. In this layer, a 3×3 convolutional kernel is used to extract local features, and the output channel size is reduced to N/2. Next, a 1×1 convolution is applied to compress the channels. This 1×1 convolution further reduces the dimensionality of the feature map, preserving information while lowering computational complexity. Then, a convolutional layer is used to restore the channel size of the feature map to *N*, using a 3×3 convolutional kernel to ensure the channel size matches the input. After each residual block’s output, an activation function (LeakyReLU) is applied to introduce non-linearity and enhance the model’s expressive ability. At the end of ResGroup1, another 1×1 convolution layer is applied to further process the feature map, with the aim of integrating and adjusting the channel information. Finally, a sigmoid activation function is applied to generate the output feature map $R_{1}$ from the first layer.

ResGroup2 has a structure identical to that of ResGroup1, with three residual blocks, each containing convolutional layers and activation operations consistent with the first layer. The output feature map of this layer is denoted as $R_{2}$.

After processing by ResGroup1 and ResGroup2, the output feature maps $R_{1}$ and $R_{2}$ are fused. First, matrix multiplication (denoted as matrix multiplication) is applied to $R_{1}$ and $R_{2}$ to extract deeper feature representations. This helps facilitate information interaction across different feature maps and promotes feature fusion. Then, the result of the matrix multiplication is added element-wise (denoted as element-wise addition) to the original input feature map $R_{in}$. This step combines the original features with the deeper processed feature maps, preserving important information from the input while integrating it with the refined features. The final output is the fused feature map $R_{out}$, which contains rich feature information that can improve the performance of subsequent tasks.

In conclusion, the ResGroup module, through multiple convolution operations and gradual feature map compression and recovery, is able to capture multi-level features from low-level to high-level, thereby enhancing the network’s expressive capability. Moreover, the design of 1×1 convolutions and matrix multiplication reduce unnecessary computations while preserving rich feature information. The LeakyReLU activation function introduces non-linearity, enabling the network to better fit complex data distributions. Furthermore, the feature fusion strategy of matrix multiplication and element-wise addition allows the ResGroup module to effectively combine features from different layers, thereby improving the overall performance of the model.

### 2.4. Loss Function

In this study, our goal is to optimize the model by minimizing a comprehensive loss function to achieve effective modeling and reconstruction of the input data. The design of the loss function takes into account multiple factors, including reconstruction error, the distribution characteristics of latent information, and the conditional entropy at different levels. The defined loss function is as follows:

(2) $$\left\{\begin{array}{c} L=\lambda D\left(x,\hat{x}\right)+H\left(\hat{z}\right),\\ H\left(\hat{z}\right)=E\left[-log_{2}\left(P_{\hat{z}}\left(\hat{z}\right)\right)\right], \end{array}\right.$$

where *L* represents the final total loss, which consists of two key components: the reconstruction error $D(x,\hat{x})$ and the entropy of the latent information $H(\hat{z})$. The parameter λ is a weighting coefficient used to adjust the relative importance between the reconstruction error $D(x,\hat{x})$ and the latent information entropy $H(\hat{z})$. When λ is large, the model focuses more on the reconstruction error, prioritizing the similarity between the output and the input, which may improve reconstruction accuracy but could compromise the learning of latent information. When λ is small, the model tends to optimize the entropy of the latent information, encouraging the learning of richer latent structures and features, thereby improving the model’s adaptability to data diversity and complexity, although it may reduce reconstruction accuracy. Since we divide the latent information into high-frequency and low-frequency components, the entropy of the latent information $H(\hat{z})$ is expressed as follows:

(3) $$H\left(\hat{z}\right)=H\left({\hat{y}}_{l}\right)+H\left({\hat{y}}_{h}\right),$$

where $H({\hat{y}}_{l})$ and $H({\hat{y}}_{h})$ represent the entropy of the low-frequency information and high-frequency information, respectively. Therefore, we define the final loss function as follows:

(4) $$\left\{\begin{array}{c} L=\lambda D\left(x,\hat{x}\right)+H\left({\hat{y}}_{l}\right)+H\left({\hat{y}}_{h}\right),\;\\ H\left({\hat{y}}_{l}\right)=E\left[-log_{2}\left(P_{{\hat{y}}_{l}|\hat{z}}\left({\hat{y}}_{l}|\hat{z}\right)\right)\right],\;\\ H\left({\hat{y}}_{h}\right)=E\left[-log_{2}\left(P_{{\hat{y}}_{h}|\hat{z}}\left({\hat{y}}_{h}|\hat{z}\right)\right)\right], \end{array}\right.$$

where the reconstruction error term $D\left(x,\hat{x}\right)$ measures the difference between the input *x* and the model output $\hat{x}$, while $H\left(\hat{z}\right)$ reflects the uncertainty in the distribution of the latent variables. To enhance the model’s ability to model information at different frequencies, we introduce the conditional entropy terms for the low-frequency part ${\hat{y}}_{l}$ and and the high-frequency part ${\hat{y}}_{h}$. These two terms represent the distributional uncertainty of the low-frequency and high-frequency components, conditioned on the latent information $\hat{z}$. By minimizing these two conditional entropy terms, we encourage the model to learn meaningful structures in both the low-frequency and high-frequency components, thereby improving the model’s adaptability to the diversity and complexity of the input data, while also avoiding overfitting to information in different frequency bands.

## 3. Experiments

Cheng2020 [23] proposed a learned image compression with discretized Gaussian mixture likelihoods and attention modules, which is the first work to achieve comparable performance of VVC in PSNR. We choose Cheng2020 [23] as the baseline of our study and evaluate the video encoding based on the ladder subband recovery and the ResGroup module with different datasets. This approach is compared with several learning-based methods, including Fu2023 [24], Zhu2022 [25], He2021 [26], Qian2022 [27], Cheng2020 [23], as well as traditional methods such as VVC and BPG.

During the training process, the ImageNet2000 dataset was used, and the images were resized to 2000×2000 pixels. This adjustment helps provide richer image details and enhances the model’s adaptability to large-sized images. To increase the diversity of the training samples and improve the generalizability of the model, a data augmentation strategy was implemented, randomly extracting 81,650 image patches of size 384×384 pixels from the resized images. Each patch was saved in a lossless PNG format to ensure no loss of image details or quality during the data augmentation process. By providing a diverse set of training samples, this step helps the model learn features from different scenes and conditions, improving its performance on unseen data.

In terms of parameter optimization, the focus was on optimizing mean squared error (MSE) and multi-scale structural similarity (MS-SSIM). For MSE, the parameter λ was selected from (0.0016, 0.0032, 0.0075, 0.015, 0.023, 0.03, 0.045) to balance compression efficiency and reconstruction precision. At lower bitrates, the number of filters *N* was set to 128 to reduce the computational burden, while at higher bitrates, *N* was set to 256 to preserve more details in the reconstructed images.

For MS-SSIM optimization, λ was selected from (12, 40, 80, 120) to help the model achieve better image quality when optimizing structural similarity. The number of filters was kept consistent with the optimization for MSE, ensuring that the network’s expressive power is fully utilized at different bitrates, thereby maximizing the structural integrity of the images.

### 3.1. Comparisons of Compression Performance

**Figure 4:**
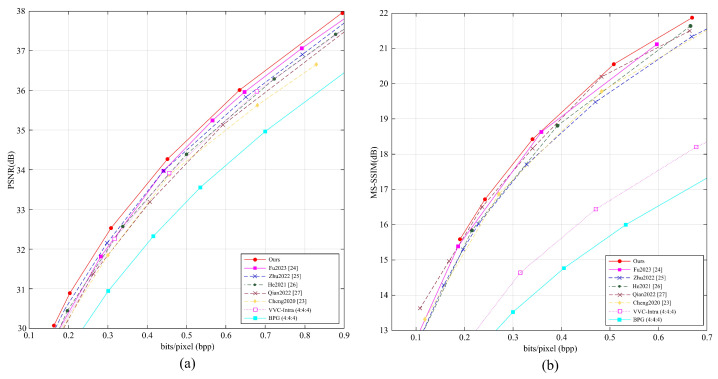
(**a**) Average PSNR and (**b**) MS-SSIM performance of different methods conducted on the Kodak test set.

In this subsection, we evaluated the average PSNR and MS-SSIM performance of different methods conducted on the Kodak dataset (all 24 images with a resolution of 768×512 are used for test), with the results shown in Figure 4. Our method was compared with several learning-based image compression methods, i.e., Fu2023 [24], Zhu2022 [25], He2021 [26], Qian2022 [27], Cheng2020 [23], as well as traditional encoding methods VVC and BPG.

Our method adopts the simple Haar transform, mainly because the Haar transform consists of two filters that separately handle high-frequency and low-frequency information. Moreover, the Haar transform not only provides good coding performance but also reduces computational complexity compared to other more complex wavelet transforms. Therefore, the Haar transform plays an important role in our LadderConv framework, further improving performance at different bitrates.

The experimental results show that at low bitrates, our method outperforms Zhu2022 [25] by about 0.2 dB in PSNR performance, and at medium to high bitrates, it outperforms Fu2023 [24] by about 0.2 dB. This indicates that our method demonstrates superior encoding performance across different bitrates.

Moreover, our method achieves the best MS-SSIM performance, further proving its superiority in visual quality. MS-SSIM, as an indicator of structural similarity, better reflects the visual quality of the image, and our approach surpasses all compared methods in this metric. The experimental results show that the LadderConv framework combined with the Haar transform can deliver higher image quality and detail recovery across different bitrates. Our method outperforms existing methods in both PSNR and MS-SSIM metrics, showing great potential for real applications.

**Figure 5:**
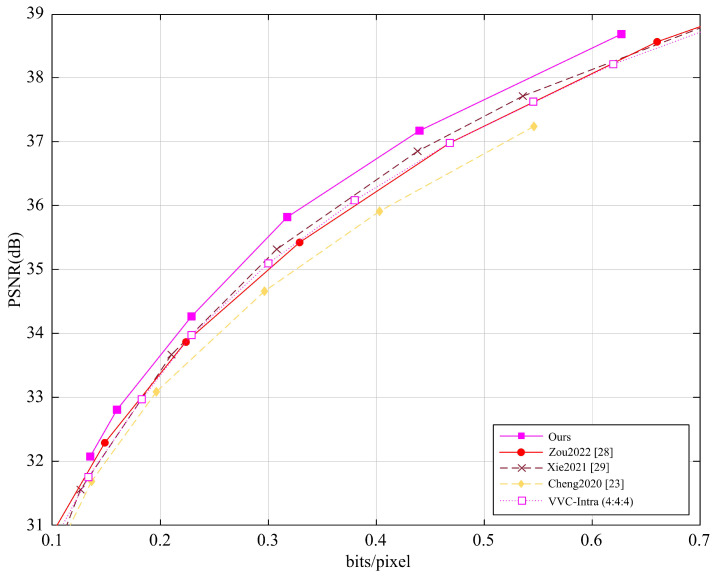
Average PSNR performance of different methods on the Tecnick dataset.

**Figure 6:**
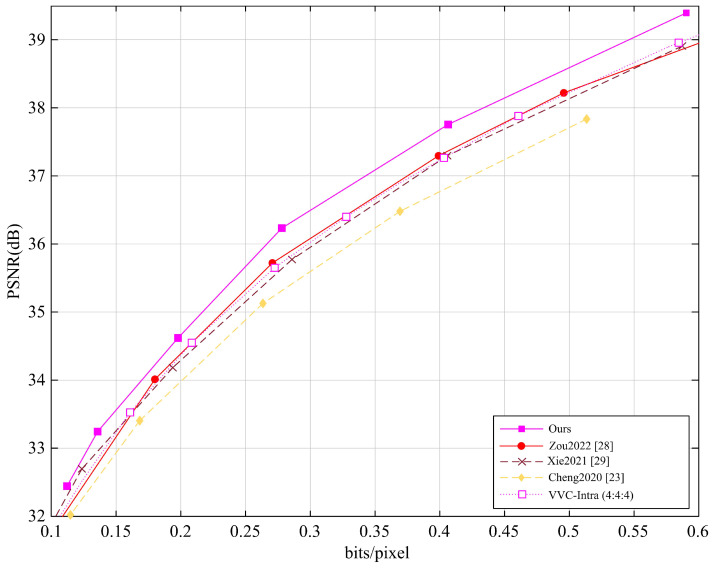
Average PSNR performance of different methods on the CLIC dataset.

Furthermore, we also evaluated the average PSNR performance of different methods on the Tecnick dataset (100 images with a resolution of 1200×1200) and the CLIC dataset (60 images with an average resolution of 2K), with the results shown in Figure 5 and Figure 6. Our method was compared with several learning-based image and video recovery methods (i.e., Zou2022 [28], Xie2021 [29], Cheng2020 [23]) and VVC.

The Tecnick and CLIC datasets cover different video content and compression complexities, i.e., the Tecnick test set contains various video scenes, while the CLIC dataset focuses on high-quality image compression. To ensure fairness, all methods were tested under the same conditions.

The experimental results show that, either on the Tecnick dataset or on the CLIC dataset, our method demonstrates notable but modest improvements in PSNR performance. For the Tecnick dataset, our method outperforms learning-based methods by approximately 0.09 dB and VVC by approximately 0.30 dB, showing an advantage in image detail recovery and quality improvement. For the CLIC dataset, our method performs about 0.11 dB better than learning-based methods and 0.25 dB better than VVC, with particularly notable performance in high compression ratio scenarios. Our method can improve image reconstruction, especially in recovering details, through multi-level feature fusion, detail recovery strategies, and efficient interaction between low-frequency and high-frequency information. The experimental results demonstrate that our method outperforms existing learning-based methods and traditional video encoding methods.

### 3.2. Comparisons of R-D Performance with Baseline

**Figure 7:**
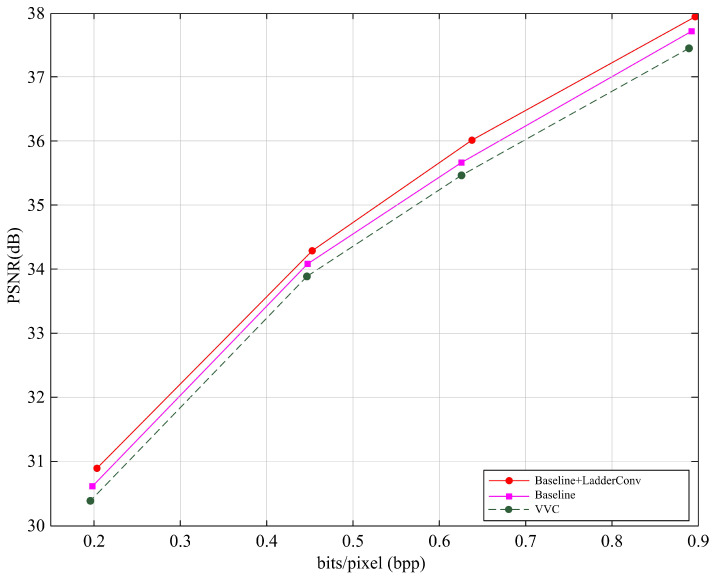
R-D performance comparison with baseline.

In this subsection, we solely compare with the baseline Cheng2020 [23] and VVC to intuitively test the performance of the proposed method. As shown in Figure 7, our proposed method demonstrates the best performance, achieving the highest PSNR value, which is approximately 0.1 dB higher than the baseline method and about 0.3 dB higher than VVC. This result clearly shows that our method has some modest advantages in image reconstruction, surpassing the traditional baseline method and VVC. Specifically, LadderConv improves image detail recovery and post-compression quality by introducing more efficient convolution operations and information transmission mechanisms, thereby achieving higher coding efficiency on the R-D performance.

## 4. Conclusions

This paper proposes an innovative video encoding framework based on DWT and ResGroup module. The framework combines the LadderConv module and the ResGroup module. Through DWT, the LadderConv module effectively decomposes the input video frame into low-frequency and high-frequency information, employing a stepwise recovery strategy to progressively reconstruct image details. It particularly demonstrates excellent performance in recovering high-frequency information, details, and textures. Experimental results show that the proposed framework outperforms both the traditional and learning-based video coding methods, especially under medium- and high-bitrate conditions, showing great potential of deep learning in video coding. Additionally, the LadderConv module integrates spatial and channel attention mechanisms, enhancing the focus on key regions and further improving the detail representation and visual quality of the reconstructed image. Experiments validate the effectiveness of this design in detail recovery, image clarity, and texture representation.

In the core design of the framework, the LadderConv module effectively enhances the reconstruction quality of image details by progressively recovering wavelet subbands. To optimize the performance of the LadderConv framework in video coding tasks, particularly in detail recovery and high-frequency information extraction, this paper introduces a novel ResGroup module. This module captures multi-level features from low level to high level through multi-layer convolution operations, along with feature map compression and recovery, enhancing the network’s expressive ability.

In the future, research will focus on further optimizing the computational efficiency and real-time processing capability of this framework, particularly in meeting the demands of low-latency video streaming transmission. Furthermore, with the continuous development of deep learning technologies, future work will explore the application of more advanced techniques in video coding, driving continuous progress and innovation in this field.

## Data Availability

The data used in this study is available from public datasets.
